# Exploring Psychological Factors and Metacognitive Beliefs in Cardiac Rehabilitation: A Single-Group Pre–Post Study

**DOI:** 10.3390/ejihpe15110236

**Published:** 2025-11-18

**Authors:** Giada Pietrabissa, Giada Rapelli, Denise Bruno, Clarissa Volpi, Lia Crotti, Gianluca Castelnuovo

**Affiliations:** 1 Clinical Psychology Research Laboratory, IRCCS Istituto Auxologico Italiano, 20149 Milan, Italy; gianluca.castelnuovo@auxologico.it; 2 Department of Psychology, Catholic University of the Sacred Heart, 20123 Milan, Italy; giada.rapelli@unicatt.it; 3 Cardiac Rehabilitation Unit, IRCCS MultiMedica, Sesto San Giovanni, 20099 Milan, Italy; denise.bruno@multimedica.it; 4 Cardiac Rehabilitation Unit, IRCCS Istituto Auxologico Italiano, 20149 Milan, Italy; c.volpi@auxologico.it (C.V.); l.crotti@auxologico.it (L.C.); 5 Department of Medicine and Surgery, Milano Bicocca University, 20126 Milan, Italy

**Keywords:** cardiac rehabilitation, cardiovascular diseases, anxiety, depression, health-related quality of life, metacognitions, clinical psychology

## Abstract

Background: Psychosocial and metacognitive factors play a critical role in cardiovascular health, influencing clinical outcomes and adherence to treatment after Cardiac Rehabilitation (CR). This study investigated the effects of an outpatient CR program on anxiety, depression, and Health-Related Quality of Life (HRQoL) in patients with established Cardiovascular Disease (CVD). Furthermore, it examined the contribution of depressive symptoms, anxiety, and dysfunctional metacognitions to patients’ perceived HRQoL. Methods: Data on demographic, psychological, and biomedical variables were obtained from 89 patients with CVD at baseline and upon completion of the CR program. Results: Participation in CR was associated with significant reductions in depressive symptoms and improvements in perceived HRQoL. Importantly, reductions in dysfunctional metacognitive beliefs emerged as the strongest predictor of post-intervention HRQoL, outweighing the predictive contribution of changes in depression and anxiety. Discussion: The results highlight the close interrelationship between psychological distress and maladaptive metacognitions, both of which are strongly associated with patients’ perceived HRQoL during CR. Conclusions: Addressing maladaptive metacognitive processes may represent a promising therapeutic target to optimize psychological adjustment and improve HRQoL in individuals undergoing CR.

## 1. Introduction

Cardiovascular Disease (CVD) remains the leading cause of mortality, morbidity, and disability in industrialized countries (Gaziano, 2022; Rittiphairoj et al., 2022). In Italy, an estimated 4.3 million individuals are affected by CVD, with a cardiovascular mortality rate of approximately 150 deaths per 100,000 inhabitants (ISTAT, 2025; Saglietto et al., 2021).

While traditional risk factors—such as age, gender, family history, hypertension, diabetes, and obesity—are well-established, they account for less than 50% of the variance in the onset and progression of CVD. For instance, a recent study reported that only about 10.9% of the variance in recurrent coronary artery disease could be attributed to traditional risk factors, highlighting the need to investigate additional determinants (Kivimäki & Steptoe, 2018).

Increasingly, research highlights the significant role of psychological factors—particularly depression and anxiety—not only in the etiopathogenesis of CVD, but also in shaping patients’ clinical outcomes, Health-Related Quality Of Life (HRQoL), and overall prognosis (Celano et al., 2018; Hare, 2021; Kivimäki & Steptoe, 2018). For instance, correlational studies have demonstrated that mood disorders, such as depression and anxiety, are associated with physiological changes, including increased heart rate and elevated blood pressure (Gorman & Sloan, 2000; Krittanawong et al., 2023; Proietti et al., 2012; Rafiei et al., 2023; Silverman et al., 2019; Suls, 2018). Moreover, psychological symptoms frequently emerge in response to the diagnosis or experience of a cardiac condition, supporting a bidirectional relationship between emotional distress and CVD (Levine et al., 2021).

In particular, systematic reviews and meta-analyses consistently conclude that depression represents a major independent risk factor for both the development and progression of CVD (Jha et al., 2019; Krittanawong et al., 2023; Rafiei et al., 2023). This association is confirmed in both men and women (Kong et al., 2022; Regitz-Zagrosek & Gebhard, 2023; Serpytis et al., 2018; Suman et al., 2023), different age groups (Li et al., 2019; Zhang et al., 2018), and across cultures (Osokpo & Riegel, 2021; Rajan et al., 2020). Anxiety disorders are also linked to the onset and progression of cardiac disease, potentially affecting heart health through various mechanisms (Celano et al., 2018; Suls, 2018).

Particularly, clinicians and researchers agree that anxiety represents a primary obstacle to the achievement of successful rehabilitation outcomes, which are crucial for secondary prevention (Lemay et al., 2019). For instance, Moser et al. (2003) revealed that anxiety disorders following a myocardial episode were associated with a variety of complications during the hospital period, such as lethal arrhythmia, permanent ischaemia, and recurrence of myocardial infarction (Moser et al., 2003). Moreover, patients with comorbid anxiety typically experience prolonged hospitalizations or extended stays in Cardiac Rehabilitation (CR) units, and report a higher symptom burden, reduced functional capacity, and poorer HRQoL (Chauvet-Gelinier & Bonin, 2017; Osuji et al., 2022; Rao et al., 2020; Spatola et al., 2018). These clinical consequences often result in increased utilization of healthcare resources and higher economic costs (Bauchner, 2019; Castelnuovo et al., 2016; Higgins et al., 2021; Palacios et al., 2018). Importantly, anxiety has also been linked to slower return to work, difficulties in resuming regular physical exercise, and challenges in re-establishing sexual activity following acute cardiac episodes (Pietrabissa et al., 2019)

In addition, psychological distress was shown to adversely affect patients’ overall well-being and HRQoL by impairing their ability to engage in daily activities, maintain employment, and participate in social interactions (Alnamrouti et al., 2025; Bahall et al., 2020; Frøjd et al., 2023; Kandasamy et al., 2025; Liao et al., 2021). A recent Italian study has further corroborated these findings, showing that psychological distress accounted for up to 37% of the variance in HRQoL, even after adjusting for clinical factors such as Body Mass Index (BMI) (Pietrabissa et al., 2024).

CR programs have consistently demonstrated effectiveness in improving cardiovascular outcomes, reducing mortality and hospitalizations, and promoting overall well-being in individuals (Mahmood et al., 2024; Myneni et al., 2024). However, the presence of symptoms of depression or anxiety may hinder adherence and reduce its effectiveness (Douma et al., 2024; Rao et al., 2020). Consequently, the assessment and management of emotional distress have become essential components of comprehensive cardiac care.

Beyond traditional psychological symptoms, recent research also highlights the relevance of metacognitive processes in the development and maintenance of mood disorders. Metacognitions, defined as beliefs about one’s own thoughts and cognitive regulation (Flavell, 1979), can perpetuate maladaptive thinking styles such as worry and rumination, thereby exacerbating anxiety and depressive symptoms (Welles & Matthews, 1996). Accordingly, several studies have demonstrated that modifying dysfunctional metacognitive beliefs can lead to a reduction in emotional distress (Lenzo et al., 2019; Wells et al., 2021). Moreover, qualitative research in CR suggests that patients’ concerns are mainly catastrophic, focusing on the risk of death and the uncertainty of recovery. These concerns are rarely linked to physical symptoms but are instead maintained by repetitive thinking patterns and attempts to make sense of the cardiac event and prevent its recurrence (McPhillips et al., 2018). At a conceptual level, this aligns with most Metacognition Questionnaire-30 (MCQ-30) domains including negative beliefs about uncontrollability/danger (NEG), perceived utility of worry (POS), and need to control thoughts (NCT). Indeed, while standard CR components (i.e., education, supervised exercise, emotional support) could plausibly attenuate NEG, POS, and NCT, effects on cognitive confidence (CC), and cognitive self-consciousness (CSC).

In this context, Metacognitive Therapy (MCT), a structured psychological intervention with established efficacy in various mental health settings, has recently shown promising results in improving emotional well-being and psychological adjustment in cardiac populations (Wells et al., 2021). These findings further suggest that targeting metacognitive dysfunctions may represent a valuable therapeutic strategy for enhancing the effectiveness of CR and optimizing patient outcomes. This literature has been cited to motivate attention to metacognitive processes in CR. Indeed, the present study did not deliver MCT or test a metacognitive intervention. Also, owing to the modest sample size, analyses were restricted to the MCQ-30 total score as a global index of metacognitive dysfunction.

Instead, this work offers—to our knowledge—the first observational examination of naturalistic changes in metacognitive beliefs during standard outpatient CR in an Italian cohort.

Specifically, the present study aims to assess whether participation in CR is associated with reductions in anxiety and depression symptoms and improvements in HRQoL and dysfunctional metacognitions among individuals with cardiovascular conditions. Additionally, it seeks to explore the extent to which anxiety, depression, and patients’ metacognitive beliefs serve as predictors of HRQoL outcomes.

Given the observational design, the following exploratory, non-causal hypotheses were formulated. First, participation in the CR program would be associated with reductions in emotional distress, improvements in HRQoL, and decreases in dysfunctional metacognitive beliefs. Second, baseline psychological and metacognitive variables would be associated with concurrent change in HRQoL.

## 2. Materials and Methods

A one-group pre–post study design was employed to determine whether the CR program was associated with a statistical improvement in psychological variables of interest (depression, anxiety, and HRQoL), and to examine the predictive role of individuals’ emotional and cognitive functioning on their patient HRQoL.

The study protocol adhered to the principles outlined in the Declaration of Helsinki and received approval from the Ethics Committee of the IRCCS Istituto Auxologico Italiano (Protocol ID: 03C202_2002).

### 2.1. Participants

Between January 2024 and July 2025, consecutive patients diagnosed with CVD and referring to the CR program at the IRCCS Istituto Auxologico Italiano, San Luca Hospital (Milan, Italy), were screened for eligibility to participate in the study within one week of their clinic intake. Potentially eligible patients were identified from clinic schedules and medical records, and their attending physician verified clinical stability prior to approach.

Trained staff then provided study information and obtained written informed consent before baseline data collection.

Participants were eligible for inclusion if they were aged 18 years or older, fluent in Italian, and had a documented history of CVD, including conditions such as myocardial infarction, coronary angioplasty, coronary artery bypass surgery, heart failure (with either reduced or preserved ejection fraction), or valvular heart disease. Only patients in a clinically stable condition were enrolled; individuals who had experienced acute cardiac events within the previous month were excluded. Additional exclusion criteria included the presence of visual impairments that could interfere with the administration of neuropsychological and psychological assessments.

All screened patients met eligibility criteria; consequently, none were excluded. Also, no refusals or research-specific dropouts were recorded, as study participation coincided with participation in the nutritional rehabilitation program and assessments were conducted as part of routine care.

### 2.2. Procedure

The cardiac rehabilitation program spans 12 weeks and consists of 20 individually tailored and professionally supervised sessions. The intervention is delivered by a multidisciplinary team comprising cardiologists, exercise physiologists, dietitians or nutritionists, and mental health professionals. Before initiating the program, patients undergo a comprehensive medical evaluation, including a review of their medical history and health behaviors. Psychological assessments are conducted at both the beginning and the end of the rehabilitation process using a standardized set of self-report questionnaires. Patients identified as experiencing elevated levels of anxiety, depression, or poor HRQoL are offered targeted non-manualized psychological support delivered by licensed psychologists within CR, including psychoeducation, adherence-focused problem-solving, and basic stress-management guidance.

### 2.3. Measures

At the beginning of CR, participants were asked to provide key demographic data, including gender, age, educational attainment, and marital and employment status. Behavioral parameters were assessed both at baseline and after program completion, encompassing sleep quality (categorized as regular or irregular), exposure to smoking (active or passive), and adherence to prescribed medication regimens, the latter evaluated at baseline through the dichotomous question: “Do you ever forget to take your medication?” (Yes/No).

Biomedical information was extracted from each patient’s clinical records at the beginning of the program. These data included CVD diagnosis, the presence of comorbid conditions such as diabetes, hypertension, dyslipidemia, vasculopathy, and obesity (defined as a BMI ≥ 30 kg/m^2^). Anthropometric and physiological measures (i.e., weight, height for BMI calculation, and blood pressure) were collected both at entry and at the end of the rehabilitation process.

In addition, psychological data were collected before and after the CR program using validated Italian versions of the following self-report questionnaires. Each assessment was administered individually in a dedicated room, with the support of a psychologist from the CR Unit.

#### 2.3.1. The Minnesota Living with Heart Failure Questionnaire (MLHFQ)

The MLHFQ (Fabbri et al., 2004) is a widely used self-report tool specifically developed to evaluate HRQoL in patients with cardiac conditions. It comprises 21 items rated on a 6-point Likert scale from 0 (not at all) to 5 (very much), assessing several physical, emotional, and socioeconomic ways heart failure can adversely affect a patient’s life. Scores are obtained by summing participants’ responses to the individual items. According to established cut-off values, a total score below 24 indicates a good HRQoL, scores between 24 and 45 reflect a moderate HRQoL, whereas scores above 45 are indicative of poor HRQoL. In the present sample, the internal consistency of the scale was good, with Cronbach’s alpha values of α = 0.825 at baseline and α = 0.907 at post-intervention.

#### 2.3.2. The Patient Health Questionnaire-9 (PHQ-9)

The PHQ-9 (Di Matteo et al., 2025) is a validated measure of depressive symptom severity, comprising 9 items scored from 0 (not at all) to 3 (nearly every day). Total scores range from 0 to 27, with cut-off values indicating: minimal/none (0–4), mild (5–9), moderate (10–14), moderately severe (15–19), and severe (20–27) depression. In this sample, the scale showed acceptable internal consistency, with Cronbach’s alpha values of α = 0.753 at baseline and α = 0.760 at post-intervention.

#### 2.3.3. The Generalized Anxiety Disorder Scale-7 (GAD-7)

The GAD-7 (Bolgeo et al., 2023) measures the severity of anxiety symptoms through 7 items scored from 0 (not at all) to 3 (nearly every day). Total scores range from 0 to 21, with cut-off values indicating: minimal/none (0–4), mild (5–9), moderate (10–14), and severe (15–21) anxiety. In the present sample, the GAD-7 demonstrated acceptable internal consistency, with Cronbach’s alpha values of α = 0.826 at baseline and α = 0.784 at post-intervention.

#### 2.3.4. The Metacognition Questionnaire-30 (MCQ-30)

The MCQ-30 (Quattropani et al., 2014) evaluates metacognitive beliefs and processes via 30 items rated on a 4-point Likert scale ranging from 1 (do not agree) to 4 (completely agree). The instrument consists of five subscales, each including six items. The Cognitive Confidence (CC) subscale (items 8, 14, 17, 24, 26, 29) measures confidence in one’s memory, attention, and cognitive abilities. The Cognitive Self-Consciousness (CSC) subscale (items 3, 5, 12, 16, 18, 30) reflects the tendency to focus on one’s own thoughts and engage in heightened self-monitoring of cognitive processes. The Positive Beliefs about Worry (POS) subscale (items 1, 7, 10, 19, 23, 28) assesses the extent to which individuals believe that worrying is useful, for instance, for problem-solving or motivation. The Negative Beliefs about Uncontrollability and Danger (NEG) subscale (items 2, 4, 9, 11, 15, 21) evaluates beliefs that worrying is uncontrollable and potentially dangerous, beliefs often associated with anxiety and distress. Last, the Need to Control Thoughts (NC) subscale (items 6, 13, 20, 22, 25, 27) measures the belief that certain thoughts must be suppressed or controlled to prevent negative outcomes. The total MCQ score is obtained by summing the scores of the individual subscales. Subscale scores range from 6 to 24; total scores range from 30 to 120, with higher scores indicating higher levels of unhelpful metacognitions.

The internal consistency of the scale was good, with Cronbach’s alpha values of α = 0.842 at baseline and α = 0.868 following CR. As for the MCQ-30 subscales, Cronbach’s alpha values at baseline and post-intervention were as follows: CC showed good reliability (α = 0.859 pre; α = 0.881 post); CSC demonstrated acceptable reliability (α = 0.689 pre; α = 0.734 post); POS also showed acceptable reliability (α = 0.684 pre; α = 0.791 post). In contrast, the NEG subscale exhibited lower internal consistency (α = 0.566 pre; α = 0.540 post), NC subscale showed moderate reliability (α = 0.682 pre; α = 0.661 post).

### 2.4. Sample Size Calculation

The sample size was calculated a priori by using the following formula provided by Tabachnick and Fidell (Tabachnick & Fidell, 2014): N > 50 + 8 m, where N is the required sample size, and m is the number of independent variables. Therefore, the sample size needed to conduct this study was at least 74 subjects.

### 2.5. Statistical Analysis

All statistical analyses were conducted using IBM SPSS Statistics for Windows, version 24.0 (IBM Corp., 2016).

Before conducting the main analyses, preliminary checks were performed to assess the assumptions for the use of parametric statistics, and the internal consistency of the questionnaires was verified using Cronbach’s Alpha coefficient (α). Descriptive statistics were calculated for all variables: continuous variables were reported as means and standard deviations, while categorical variables were summarized using frequencies and percentages. Categorical changes in MLHFQ (HRQoL), PHQ-9 (depressive symptoms), GAD-7 (anxiety symptoms) scores were computed based on established clinical cut-offs to facilitate interpretation of clinical significance. These data are presented for descriptive purposes only and were not subjected to inferential testing. Moreover, pre–post change was assessed via the Reliable Change Index (RCI) for four outcomes—MLHFQ, PHQ-9, GAD-7, and MCQ-30 (dysfunctional metacognitions). RCI was classified as reliable improvement (RCI ≤ −1.96), no reliable change (−1.96 < RCI < 1.96), or reliable deterioration (RCI ≥ 1.96).

In addition, bivariate associations between continuous variables were examined using Spearman’s rank correlation coefficient.

Subsequently, change scores (Δ) were computed for depressive symptoms, anxiety symptoms, and dysfunctional metacognitive beliefs, and were entered as predictors in a three-step hierarchical multiple linear regression, with post-intervention HRQoL as the dependent variable. In Step 1, depression was entered as the sole predictor; in Step 2, anxiety was added alongside depression; and in Step 3, metacognitive beliefs were also included. Although some variables were not normally distributed, hierarchical multiple linear regression was performed, as this method is considered robust to moderate violations of normality—especially with sample sizes exceeding 30 participants (Knief & Forstmeier, 2021). To verify model validity, residuals were inspected for normality, linearity, and homoscedasticity, and variance inflation factors (VIFs) were calculated to rule out multicollinearity (Pek et al., 2018). Potential methods for handling missing data (i.e., pairwise deletion for correlational analyses and the use of paired observations for pre–post comparisons) were employed.

## 3. Results

### 3.1. Baseline Characteristics of the Sample

The study sample consisted of 89 participants, including 27 women (30.3%) and 62 men (69.7%). The mean age was 68.46 years (SD = 9.69; range = 30–88), and the mean BMI was 25.56 kg/m^2^ (SD = 3.72; range = 19–40).

Regarding education, 2.2% of participants had completed primary school, 12.4% middle school, 24.7% high school, 43.8% held a bachelor’s degree, 9.0% a master’s degree, and 7.9% a Ph.D. In terms of marital status, the majority were married or cohabiting (76.4%), while 9.0% were unmarried, 11.2% separated or divorced, and 3.4% widowed. With respect to employment, more than half were retired (58.4%), followed by employees (15.7%), self-employed workers (15.7%), housewives (3.4%), unemployed (4.5%), and other occupations (2.2%).

The most common diagnosis was myocardial infarction (58.4%), followed by coronary angioplasty (9.0%), coronary artery bypass surgery (9.0%), heart failure (8.0%), valve replacement (9.0%), and valve repair (7.0%). Comorbid conditions included hypertension (58.4%), dyslipidemia (56.2%), diabetes (28.1%), vasculopathy (19.5%), and obesity (9.0%).

Most participants (74.2%) reported adherence to prescribed medication, while 25.8% did not adhere to treatment. Sleep quality was reported as regular by 82% of the sample and irregular by 18%. Only 8% of participants were current smokers, whereas 92% reported not smoking.

The mean score on MLHFQ, a measure of HRQoL, was 15.85 (SD = 14.31), with scores ranging from 0 to 55. Depressive symptoms, as assessed by the PHQ-9, yielded a mean score of 3.63 (SD = 3.29), with a range of 0 to 13. Anxiety levels, measured using the GAD-7, had a mean of 3.25 (SD = 3.20), with a range of 0 to 18. Metacognitive beliefs and processes, evaluated through the MCQ-30, showed an overall mean score of 53.47 (SD = 10.91), with total scores ranging from 30 to 95. Regarding the subscales of the MCQ-30, the mean scores were as follows: lack of cognitive confidence (M = 9.75; SD = 4.00), cognitive self-consciousness (M = 13.95; SD = 3.96), Positive Beliefs about Worry (M = 9.32; SD = 2.98), negative beliefs about uncontrollability and danger (M = 10.73; SD = 3.06), and need to control thoughts (M = 9.72; SD = 3.45). A detailed description of the sample characteristics is reported in Table 1.

### 3.2. Clinical Significance of Changes in Psychological Factors and Metacognitive Beliefs Following Cardiac Rehabilitation

Based on the clinical cut-off scores for each outcome measure, categorical changes from pre- to post-rehabilitation were reported to provide a clearer view of the clinical significance of outcomes. These values were intended for descriptive interpretation only and were not analyzed statistically.

An improvement in health-related quality of life (MLHFQ) was observed, with the proportion of participants classified as having “Good” HRQoL increasing from 68 to 75 individuals. The number of participants in the “Moderate” category decreased from 16 to 9, while those with “Poor” HRQoL remained stable at 5.

Concerning depressive symptoms (PHQ-9), most participants were initially classified as having no depressive symptoms (“None”), increasing from 65 pre-intervention to 71 post-intervention. Those with mild depression decreased from 17 to 13, and those with moderate depression slightly decreased from 7 to 5. No participants were classified as having moderately severe or severe depression at either time point.

For anxiety symptoms (GAD-7), the majority of participants were classified as having minimal anxiety, increasing from 64 pre-intervention to 70 post-intervention. Participants with mild anxiety decreased from 22 to 16, and those with moderate anxiety increased slightly from 2 to 3. One participant was initially classified as having severe anxiety, but no participants remained in this category post-intervention.

Last, regarding the MCQ-30, although no clinical cut-offs are established, the group-level reduction in mean scores after the intervention suggests improved metacognitive functioning, with participants endorsing fewer unhelpful metacognitions about their emotional experiences.

Still, in Reliable Change Index (RCI) analyses, most participants showed no reliable change across measures. For PHQ-9, 8/89 (9.0%) met reliable improvement, 80/89 (89.9%) showed no reliable change, and 1/89 (1.1%) showed reliable deterioration; for GAD-7, the corresponding figures were 8/89 (9.0%), 76/89 (85.4%), and 5/89 (5.6%); for MLHFQ, 14/89 (15.7%), 68/89 (76.4%), and 7/89 (7.9%); and for MCQ-30 total (MQ), 17/80 (21.2%), 51/80 (63.7%), and 12/80 (15.0%). These patterns indicate that reliable improvement was most frequent for metacognitions (MCQ-30) and HRQoL (MLHFQ), while a minority exhibited reliable deterioration—more often on the MCQ-30—underscoring the need for cautious interpretation of individual-level change (see Supplementary Table S1). Pre–post reliable change estimates for all outcome measures are reported in Table 2.

### 3.3. Associations Between Psychological Variables, Metacognitions, and HRQoL

Spearman’s rank-order correlation analyses were conducted to explore the associations among baseline levels of HRQoL, depressive symptoms, anxiety, and dysfunctional metacognitive beliefs (Table 3).

Results showed that greater impairment in HRQoL, as measured by the MLHFQ, was moderately and significantly associated with higher levels of depressive symptoms (PHQ-9; ρ = 0.552, *p* < 0.01), anxiety (GAD-7; ρ = 0.404, *p* < 0.01), and more dysfunctional metacognitive beliefs (MCQ-30; ρ = 0.238, *p* < 0.05). In addition, depressive symptoms were moderately correlated with anxiety (ρ = 0.599, *p* < 0.01) and showed a weaker but significant relationship with dysfunctional metacognitive beliefs (ρ = 0.259, *p* < 0.05). Similarly, anxiety symptoms were weakly but significantly related to maladaptive metacognitions (ρ = 0.274, *p* < 0.05).

### 3.4. Hierarchical Multiple Regression Predicting Post-Intervention HRQoL

A three-block hierarchical multiple regression was performed to examine the associations between change scores (Δ) in depressive symptoms (PHQ-9), anxiety (GAD-7), and dysfunctional metacognitive beliefs (MCQ-30) and post-CR HRQoL. Despite the non-normal distribution of some independent variables, residual diagnostics indicated values within acceptable ranges (standardized residuals between −1.6 and +3.3) and no substantial multicollinearity issues (all VIFs < 2), thus satisfying the assumptions of linear regression.

At Step 1, change in depressive symptoms accounted for 6.4% of the variance (R^2^ = 0.064; adj. R^2^ = 0.052), with ΔPHQ-9 showing a positive association with MLHFQ (B = 1.233, SE = 0.531, β = 0.253, 95% CI [0.177, 2.290], *p* = 0.023).

At Step 2, the addition of change in anxiety symptoms (ΔGAD-7) did not significantly improve model fit (ΔR^2^ ≈ 0.000, ΔF(1, 78) = 0.012, *p* = 0.912), and both ΔGAD-7 (B = 0.071, SE = 0.635, β = 0.017, *p* = 0.912) and ΔPHQ-9 (B = 1.179, SE = 0.727, *p* = 0.109) were not statistically significant.

At Step 3, the inclusion of change in dysfunctional metacognitive beliefs (ΔMCQ-30) was associated with lower MLHFQ scores (B = −0.377, SE = 0.124, β = −0.331, 95% CI [−0.624, −0.130], *p* = 0.003) and a modest increase in explained variance (ΔR^2^ = 0.100; ΔF(1, 77) = 9.257, *p* = 0.003). The final model explained 16.5% of the variance (adj. R^2^ = 0.132; F(3, 77) = 5.057, *p* = 0.003); ΔPHQ-9 (B = 1.066, SE = 0.692, *p* = 0.128) and ΔGAD-7 (B = 0.540, SE = 0.624, *p* = 0.389) were not significant. These results should be interpreted as associations within a single-group, pre–post design, with overall effect sizes that are modest (Durbin–Watson = 2.197). Results are displayed in Table 4 below.

## 4. Discussion

This study was designed to evaluate the impact of a CR program on reducing anxiety and depressive symptoms while enhancing HRQoL and fostering more adaptive metacognitive processes among individuals with CVD. A further objective was to examine the associations of psychological distress and maladaptive metacognitive beliefs with HRQoL outcomes.

In line with previous evidence, participation in CR was related to reductions in depressive symptoms and improvements in perceived HRQoL (Pietrabissa et al., 2017; Pietrabissa et al., 2021; Spatola et al., 2024; Spatola et al., 2025).

Reductions in dysfunctional metacognitions emerged as the strongest correlate of post-intervention HRQoL, exceeding the contribution of changes in depressive and anxiety symptoms. This pattern tentatively underscores the relevance of metacognitive processes for patients’ adjustment and HRQoL and suggests that addressing maladaptive metacognitions could be a promising target within integrative CR programs.

In this study, the sample comprised primarily older male participants, which aligns with the known epidemiology of CVD (Benjamin et al., 2019), as these conditions are more prevalent among men and older adults. Educational attainment within the cohort was relatively high, with more than 60% of participants holding at least a bachelor’s degree. Additionally, most participants were married or cohabiting (76.4%). This is noteworthy, as these factors are commonly linked to stronger treatment adherence and more favorable psychological outcomes in patients with chronic conditions, including CVD (Hu et al., 2024; Rapelli et al., 2024, 2021, 2022, 2023; Xu et al., 2025).

Accordingly, health behaviors within the sample appeared encouraging. At baseline, the majority of participants reported regular sleep quality (82%) and good adherence to prescribed medications (74.2%), while only a small proportion identified as current smokers (8%). These findings are further supported by recent studies showing that older adults with higher educational attainment and stronger social support are more likely to adopt and maintain health-promoting behaviors, including consistent medication adherence, reduced smoking, and healthier lifestyle routines (Kalantarzadeh et al., 2022; Nebel et al., 2025; Tao et al., 2025; Xu et al., 2025).

The clinical profile of the participants was consistent with expectations for a cardiovascular population. Myocardial infarction emerged as the most common diagnosis, affecting more than half of the individuals (58.4%). In addition, comorbidities such as hypertension (58.4%), dyslipidemia (56.2%), and diabetes (28.1%) were highly prevalent, reflecting the well-documented clustering of cardiovascular risk factors typically observed in this patient group (Einarson et al., 2018; Roth et al., 2020). Also, the sample’s mean BMI (25.56 kg/m^2^) is consistent with prior findings from similar CR cohorts, in which overweight is a common risk factor for adverse cardiovascular outcomes (Pietrabissa et al., 2023, 2017).

Concerning psychological functioning, baseline findings indicated that participants, on average, reported relatively low levels of depressive and anxiety symptoms, suggesting a generally favorable emotional profile within the sample. Still, metacognitive beliefs and processes, as measured by the MCQ-30, revealed high scores, particularly in the subscales of cognitive self-consciousness and negative beliefs about the uncontrollability and danger of thoughts.

As previously discussed, these findings appear consistent with prior research suggesting potential benefits CR on HRQoL and emotional well-being, although the mechanisms underlying these effects remain to be fully clarified.

For example, a 2019 meta-analysis found that exercise-based CR significantly reduces both anxiety and depressive symptoms in post-myocardial infarction patients (Zheng et al., 2019). A systematic review and meta-analysis (2024) further confirmed that CR leads to sustained enhancement in the physical component of HRQoL up to six months after program initiation (Moreira et al., 2024). In addition, CR that includes psychologically enhanced components has been shown to lower resting blood pressure and improve overall HRQoL (Wrzeciono et al., 2024). Although improvements in anxiety did not reach statistical significance, the observed trend mirrors previous reports highlighting that reductions in anxiety symptoms during CR are often less robust and may require additional, targeted psychological interventions (Wells et al., 2018).

This suggests that, while depressive symptoms may respond more uniformly, anxiety outcomes could be potentially moderated by or depends on individual differences, including maladaptive cognitive styles characterized by pervasive negative thinking (Spinhoven et al., 2018). Indeed, recent systematic reviews highlighted that dysfunctional metacognition is significantly correlated with increased levels of anxiety and depression, and is notably associated with lower perceived HRQoL across a variety of chronic illnesses (Capobianco et al., 2020; Lenzo et al., 2019). Furthermore, empirical findings from Havnen and colleagues (Havnen et al., 2024) suggest that more severe dysfunctional metacognitive beliefs are associated with a measurable decline in HRQoL, reinforcing the detrimental cognitive-emotional impact of these beliefs (Havnen et al., 2024).

A notable observation in this sample is the naturalistic reduction in dysfunctional metacognitive beliefs from pre- to post-CR. Although CR primarily targets physical and lifestyle outcomes, such changes may reflect indirect effects of routine components Changes in metacognitions were also associated with post-intervention HRQoL and remained the strongest correlate after accounting for changes in depressive and anxiety symptoms, whose associations were attenuated and non-significant. This supports the theoretical model proposed by Wells (2011), which emphasizes the role of metacognitive processes in the maintenance of psychological distress (Wells, 2011). It also supports the view that dysfunctional metacognitions may act as transdiagnostic mechanisms influencing emotional regulation (Normann & Morina, 2018). Importantly, this work provides preliminary evidence from an Italian outpatient CR setting, a context underrepresented in the literature, and thus offers hypothesis-generating data to prioritize metacognitive processes as candidates for targeted, integrative CR enhancements.

### 4.1. Clinical Implications

From a clinical perspective, these results suggest that monitoring and addressing metacognitive processes during CR could enhance patients’ psychological recovery and overall HRQoL. Tailored interventions such as MCT or brief cognitive-behavioral modules focusing on worry, rumination, and beliefs about the uncontrollability of thoughts could be integrated into CR programs. Such interventions may provide additional benefit beyond the standard focus on exercise and lifestyle modification, particularly for patients presenting with psychological vulnerability.

### 4.2. Limitations and Future Directions

Some limitations of this study should be acknowledged. First, the use of a single-group pre–post design without a control group constrains internal validity. Observed changes cannot, therefore, be attributed solely to CR, and pre–post differences should be interpreted as associations rather than causal effects.

A further limitation is that potential confounders were not adjusted for (e.g., receipt of complementary psychological support, clinical-severity indices, comorbidities, medication use, demographic factors, and subscale-level variation), so residual confounding cannot be excluded. Still, given the modest sample, this would have reduced statistical power and increased the risk of model overfitting. Accordingly, estimates must be interpreted as associative, and residual confounding cannot be ruled out. In addition, although missing data were minimal and handled using pre-specified procedures, residual cannot be entirely ruled out.

Even so, observational data provide valuable insights into how interventions perform under routine clinical conditions. Nonetheless, reliance on self-report measures may have introduced response bias or social desirability effects. Also, the absence of validated MCQ-30 clinical cut-offs limits inferences about the clinical relevance of changes at the individual level. However, a strength of the present study is the use of a disease-specific measure of HRQoL, namely the Minnesota Living with Heart Failure Questionnaire (MLHFQ), which is widely recognized for its reliability and sensitivity in capturing the perceived HRQoL of patients with CVD.

Moreover, the relatively low average levels of anxiety and depression suggest that the sample may not fully represent patients experiencing more severe psychological distress, thereby limiting the generalizability of the findings to more clinically impaired populations. In addition, the potential influence of response or social desirability bias due to the use of self-report measures cannot be entirely ruled out.

Another consideration is the absence of follow-up assessments, which prevents evaluation of the long-term sustainability of the observed improvements in both physical and psychological domains.

Future studies should adopt adequately powered, controlled studies to test domain-specific hypotheses and mechanisms, include long-term follow-up assessments and objective physiological or behavioral measures alongside self-reports, and evaluate whether integrating targeted psychological or metacognitive interventions within CR protocols further enhance outcomes.

## 5. Conclusions

This study provides preliminary evidence that outpatient CR may be associated with improvement in HRQoL and depressive symptoms, and may reduce dysfunctional metacognitive beliefs, which appeared to be the strongest predictor of post-rehabilitation HRQoL. However, given the pilot nature and limitations of the present study, these results should be viewed as hypothesis-generating, and the potential value of targeting metacognitive processes within CR requires confirmation in more rigorous studies.

## Figures and Tables

**Table 1:** Sample characteristics at baseline.

Variable	*n*	%	
Gender			
Female	27	30.3	
Male	62	69.7	
Education			
Primary school diploma	2	2.2	
Middle school diploma	11	12.4	
High school diploma	22	24.7	
Bachelor’s degree	39	43.8	
Master’s degree	8	9.0	
Ph.D.	7	7.9	
Civil status			
Unmarried	8	9	
Married/cohabiting	68	76.4	
Separated/divorced	10	11.2	
Widow/widower	3	3.4	
Job			
Employee	14	15.7	
Self-employed	14	15.7	
Housewife	3	3.4	
Unemployed	4	4.5	
Retired	52	58.4	
Other	2	2.2	
Diagnosis			
Myocardial Infarction	52	58.4	
Coronary angioplasty	8	9	
Coronary artery bypass	8	9	
Heart failure	7	8	
Valve replacement	8	9	
Valve plastic	6	7	
Comorbidities			
Hypertension	52	58.4	
Dyslipidemia	50	56.2	
Diabetes	25	28.1	
Vasculopathy	17	19.5	
Obesity	8	9%	
Adherence to medication			
Yes	66	74.2	
No	23	25.8	
Sleep quality			
Irregular	16	18	
Regular	73	82	
Smoke			
Yes	7	8	
No	82	92	
	**Mean**	**SD**	**Range**
Age (years)	68.46	9.687	30–88
BMI (kg/m^2^)	25.56	3.715	19–40
MLHFQ	15.85	14.314	0–55
PHQ-9	3.63	3.294	0–13
GAD-7	3.25	3.199	0–18
MCQ-30	53.47	10.91	30–95
MCQ-30_CC	9.75	4.002	5–24
MCQ-30_CSC	13.95	3.956	6–24
MCQ-30_POS	9.32	2.978	6–18
MCQ-30_NEG	10.73	3.058	6–21
MCQ-30_NC	9.72	3.454	5–22

Notes: BMI: Body Mass Index; MLHFQ: Minnesota Living with Heart Failure Questionnaire; PHQ-9: Patient Health Questionnaire-9; GAD-7: Generalized Anxiety Disorder Scale-7; MCQ-30: Metacognition Questionnaire-30; CC: Lack of Cognitive Confidence; CSC: Cognitive Self-Consciousness; POS: Positive Beliefs about Worry; NEG: Negative Beliefs about Uncontrollability and Danger; NC: Need to Control thoughts.

**Table 2:** Pre–Post Reliable Change by Outcome Measure.

	PHQ-9		GAD-7		MLHFQ		MCQ	
Category	n	%	n	%	n	%	n	%
Reliable improvement	8	9	8	9	14	15.7	17	21.2
No reliable change	80	89.9	76	85.4	68	76.4	51	63.7
Reliable deterioration	1	1.1	5	5.6	7	7.9	12	15

Notes: PHQ-9: Patient Health Questionnaire-9; GAD-7: Generalized Anxiety Disorder Scale-7; MLHFQ: Minnesota Living with Heart Failure Questionnaire; MCQ-30: Metacognition Questionnaire-30.

**Table 3:** Descriptive statistics and Correlation between variables.

	Descriptive				Correlations			
	Mean	SD	SK	K	MLHFQ	PHQ-9	GAD-7	MCQ-30
MLHFQ	15.85	14.31	1.23	0.76	-			
PHQ-9	3.63	3.29	1.24	0.96	0.552 **	-		
GAD-7	3.25	3.20	1.81	5.14	0.404 **	0.599 **	-	
MCQ-30	53.47	10.92	1.05	2.11	0.238 *	0.259 *	0.274 *	-

Notes: * *p* < 0.05; ** *p* < 0.01. MLHFQ: Minnesota Living with Heart Failure Questionnaire; PHQ-9: Patient Health Questionnaire-9; GAD-7: Generalized Anxiety Disorder Scale-7; MCQ-30: Metacognition Questionnaire-30.

**Table 4:** Model Summary of the Hierarchical Multiple Regression.

Model	Predictor	B	SE 95% CI	β	t	*p*	R	R^2^	Adj. R^2^	ΔR^2^	F (df)	Durbin–Watson
Step 1	ΔPHQ-9	1.233	0.531 [0.177, 2.290]	0.253	2.325	0.023	0.253	0.064	0.052	0.064	5.404 (1, 79)	
Step 2	ΔPHQ-9	1.179	0.727 [−0.269, 2.626]	0.242	1.621	0.109	0.253	0.064	0.040	0.000	2.675 (2, 78)	
	ΔGAD-7	0.071	0.635 [−1.194, 1.336]	0.017	0.111	0.912						
Step 3	ΔPHQ-9	1.066	0.692 [−0.313, 2.445]	0.219	1.539	0.128	0.406	0.165	0.132	0.100	5.057 (3, 77)	2.197
	ΔGAD-7	0.540	0.624 [−0.701, 1.782]	0.127	0.867	0.389						
	ΔMAQ-30	−0.377	0.124 [−0.624, −0.130]	−0.331	−3.043	0.003						

Notes: Model = hierarchical block (step); Predictor = independent variable. B = unstandardized regression coefficient; SE = standard error of B; 95% CI = 95% confidence interval for B; β = standardized coefficient; t = t statistic for B; *p* = two-tailed significance level; R = multiple correlation for the model; R^2^ = proportion of variance explained; Adj. R^2^ = R^2^ adjusted for degrees of freedom; ΔR^2^ = change in R^2^ from the previous model; F (df) = omnibus F test of model fit with numerator and denominator degrees of freedom; Durbin–Watson = statistic testing first-order autocorrelation of residuals (≈2 indicates independence). Predictors were entered hierarchically: Step 1 = ΔPHQ-9 (change in depressive symptoms); Step 2 adds ΔGAD-7 (change in anxiety); Step 3 adds ΔMCQ-30 total (change in metacognitive dysfunction). Positive B values indicate associations with higher MLHFQ (worse HRQoL), negative B values indicate associations with lower MLHFQ (better HRQoL).

## Data Availability

Data are available upon reasonable request.

## Supplementary Materials

The following supporting information can be downloaded at: https://www.mdpi.com/article/10.3390/ejihpe15110236/s1, Table S1: Pre–Post Reliable Change Indices.
